# Respiratory muscle strength can improve the prognostic assessment in COPD

**DOI:** 10.1038/s41598-024-54264-w

**Published:** 2024-05-29

**Authors:** Rebeca Nunes Silva, Cássia da Luz Goulart, Claudio R. de Oliveira, Renata Gonçalves Mendes, Ross Arena, Jonathan Myers, Audrey Borghi-Silva

**Affiliations:** 1 Department of Physiotherapy, Cardiopulmonary Physiotherapy Laboratory (LACAP), Federal University of São Carlos (UFSCar), São Carlos, São Paulo 13565-905 Brazil; 2 Department of Medicine, Federal University of São Carlos (UFSCar), São Carlos, São Paulo Brazil; 3 Department of Physical Therapy, College of Applied Health Sciences, University of Illinois Chicago, Chicago, IL USA; 4 Veterans Affairs Palo Alto HealthCare System, Stanford University, Palo Alto, CA USA

**Keywords:** Respiration, Chronic obstructive pulmonary disease

## Abstract

Impaired lung function, respiratory muscle weakness and exercise intolerance are present in COPD and contribute to poor prognosis. However, the contribution of the combination of these manifestations to define prognosis in COPD is still unknown. This study aimed to define cut-off points for both inspiratory and expiratory muscle strength (MIP and MEP, respectively) for mortality prediction over 42-months in patients with COPD, and to investigate its combination with other noninvasive established prognostic measures (FEV_1_, *V̇*O_2peak_ and 6MWD) to improve risk identification. Patients with COPD performed pulmonary function, respiratory muscle strength, six-minute walk and cardiopulmonary exercise tests, and were followed over 42 months to analyze all-cause mortality. A total of 79 patients were included. The sample was mostly (91.1%) comprised of severe (*n* = 37) and very severe (*n* = 34) COPD, and 43 (54%) patients died during the follow-up period. Cut-points of ≤ 55 and ≤ 80 cmH_2_O for MIP and MEP, respectively, were associated with increased risk of death (log-rank *p* = 0.0001 for both MIP and MEP) in 42 months. Furthermore, MIP and MEP substantially improved the mortality risk assessment when combined with FEV_1_ (log-ranks *p* = 0.006 for MIP and *p* < 0.001 for MEP), *V̇*O_2peak_ (log-rank: *p* < 0.001 for both MIP and MEP) and 6MWD (log-ranks: *p* = 0.005 for MIP; *p* = 0.015 for MEP). Thus, patients severely affected by COPD presenting MIP ≤ 55 and/or MEP ≤ 80 cmH2O are at increased risk of mortality. Furthermore, MIP and MEP substantially improve the mortality risk assessment when combined with FEV1, V̇O2peak and 6MWD in patients with COPD.

## Introduction

Due to the progressive pathophysiology of chronic obstructive pulmonary disease (COPD), prognostic assessments to identify patients at higher risk of exacerbations, hospitalizations and death has received considerable attention, especially when dealing with severe patients who are more prone to lung volume reduction surgery or lung transplantation^1^. Previous findings show that impaired lung function^2^, respiratory muscle weakness^3^ and exercise intolerance^4^ are strong and independent predictors for mortality and can be useful in clinical practice to identify patients at increased risk for poor prognosis, such as hospitalization and death^1^.

In this sense, lung function is assessed through spirometry and the forced expired volume in the first second of the maneuver (FEV_1_) is the standard parameter to classify the obstruction severity in COPD^1^. The respiratory muscle weakness is usually assessed through maximum inspiratory (MIP) and expiratory (MEP) muscles pressures^5^, and although MEP was less investigated, MIP has been shown to predict all-cause mortality in COPD^3^. The exercise intolerance is assessed through the cardiopulmonary exercise test (CPET)^6^, but because its application is hindered by the added cost and expertise, the six-minute walk test (6MWT) is widely used in clinical practice^1^, and despite it does not assess the exercise capacity, 6MWT allows to assess the functional performance, which is also an independent predictor of mortality in COPD^1^.

Thus, because risk stratification can guide the management of the disease, and because lung function, respiratory muscle strength, exercise capacity and functional performance are all related to the prognosis of COPD^2,3,7–10^, investigating their interactions and impact over time can improve the identification of patients at higher risk of mortality. Moreover, evidence suggests that MIP can improve the risk stratification when associated to peak oxygen uptake (*V̇*O_2peak_) in patients with heart failure (HF)^11^, and although the interactions with MEP has not been investigated, literature shows that the expiratory muscles also play an important role in the mechanics of ventilation, and its improvement can lead to a multitude of benefits^10^.

Therefore, given the importance of the prognosis assessment for an accurate management of COPD, and understanding the lack of studies evaluating MEP, the first objective of this study was to identify cut-off points for MIP and MEP to predict all-cause mortality risk in patients with COPD, and because the interaction of MIP with another established prognostic measures seems to improve the risk stratification in another populations^11^ but is less investigated in COPD, the second objective was to investigate the combination of both MIP and MEP with FEV_1_, *V̇*O_2peak_ and six-minute walk distance (6MWD), which are known independent predictors of mortality in COPD. We hypothesized that MIP and MEP cut-off values derived from this analysis would be independent predictors of mortality, and that mortality risk assessment of patients severely affected by COPD would be improved by MIP and MEP when associated with other established prognostic measures.

## Methods

### Study design and sample

In this retrospective cohort study, patients with COPD^1^ were selected from a sample derived from a previous study conducted from February 2002 to February 2008 (Protocol #025/2002), from which all participants provided written informed consent and were followed over 42 months. The patients included in this analysis were attending to the Pneumology ambulatory at the Federal University of São Carlos (UFSCar). At that moment, the subjects were selected to be included in a clinical study assessing patients with COPD with moderate-to-very severe obstruction^12^, contemplating the severely affected patients included in this study. This retrospective analysis was approved by the Institutional Research Ethics Committee (Protocol #91,088,318.7.1001.5504) from the Federal University of São Carlos, and all methods performed are in accordance with the Declaration of Helsinki for medical research involving human subjects.

All participants were clinically stable for at least 3 months and receiving optimized medical management. Exclusion criteria consisted of (1) musculoskeletal or neurological disorders affecting the ability to exercise; (2) exacerbation requiring hospitalization three months before the study; (3) diagnosis of malignant disease; (4) implantable pacemaker; (5) myocardial infarction (≤ three months before the study); and (6) complex cardiac arrhythmias.

## Study protocol

Every participant completed the assessments in three days, consisting of the following: **visit 1**: clinical evaluation, followed by both pulmonary function (PF) and respiratory muscle strength (RMS) tests; **visit 2:** 6MWT; and **visit 3**: CPET. Participants were instructed to abstain from caffeine products and strenuous activities within 24 h prior to the evaluations.

## Measurements

### Pulmonary function and respiratory muscle strength

Spirometry (Masterlab Pro; Jaeger; Würzburg, Germany) was performed and interpreted according to the standards established by the *American Thoracic Society* and *European Respiratory Society*^13^ by the same trained evaluator in all participants to assess PF. To classify the severity of the disease, post-bronchodilator criteria was used according to GOLD^1^, and predicted values were calculated according to the Brazilian population^14^.

A digital manometer (MDI, MVD300, Brazil) was used to assess RMS. MIP was obtained after the individual expired to residual volume and perform a maximal effort inspiration against a closed valve for two seconds, during which the pressure was measured. For the MEP assessment, patients underwent an inspiration to total lung capacity, followed by a maximal effort expiration against a closed valve for two seconds^15^.

### Six-minute walk test (6MWT)

Subjects were instructed and encouraged to walk the longest possible distance in six minutes on a 30-m-long flat corridor, previously demarked by a colored tape. The 6MWT was performed according to the *American Thoracic Society* recommendations^16^.

### Cardiopulmonary exercise test (CPET)

Breath-by-breath ventilatory data was obtained throughout the test using an ergospirometer (Oxycon Mobile Mijnhardt/Jäger, Germany), and the following data were recorded or calculated based on the recorded data: oxygen uptake (*V̇*O_2_), carbon dioxide output (*V̇*CO_2_), ventilation (*V̇*_E_,), respiratory exchange ratio (RER), oxygen uptake efficiency slope (OUES), ventilatory efficiency up to peak exercise (the *V̇*_E_/*V̇*CO_2slope_) and breathing reserve (*V̇*_E_/VVM).

Incremental ramp adjustment of work rate was individually selected using an upright cycle ergometer. The load increment was individually selected between 5 and 10 W/min based on the dyspnea reported by the patient during physical activity and the experience of the research team, to achieve maximal effort within 8–12 min^6^. The gas analyzer had volume and gas calibrated immediately before each test as per manufacturer specifications.

The CPET protocol was composed of the following stages: 1) 5 min of rest; 2) 1 min warm-up of unloaded cycling at 60 rotations per minute (rpm); 3) incremental phase (5–10 W/min, ramp protocol); 4) 1-min active cool-down of unloaded cycling; and 5) 5-min passive cool-down at rest. A twelve-lead electrocardiogram was continuously monitored throughout the test (WinCardio, Micromed, Brazil), which was terminated when the participant pedaled at their maximum possible effort level or exhibited an established termination criteria such as angina, electrocardiographic evidence of ischemia, or malignant arrhythmias^6^.

### Follow-up assessment

Participants were followed for 42 months by telephone calls to their home or family physician after the first evaluation. The main outcome assessed was all-cause mortality.

### Statistical analysis

SPSS® version 25 was used for data analyses, and p-values < 0.05 were considered significant. *Kolmogorov–Smirnov test* verified data distribution. To compare survivors *versus* non-survivors, *Students’ t-test* and *Mann–Whitney test* was used depending on the data distribution.

*Receiver operating characteristic (ROC) curve* was used to identify the cut-off points of MIP and MEP in determining mortality risk. 95% confidence intervals (CI) and a lower limit greater than 0.50 were considered, and the cut-off points of the variables that obtained significant areas under the curve were identified, with their respective values of sensitivity and specificity.

Survival probability was analyzed through the *Kaplan–Meier* method. The impact of MIP, MEP, FEV_1_% of predicted, *V̇*O_2peak_, and 6MWD, as well as of both MIP and MEP associated with FEV_1_ using a previously described range of 36–55% of predicted^2^, *V̇*O_2peak_ using a previously reported range of 10–20 ml/kg^/^min^11^ and 6MWD using a defined range of 250–550 m was analyzed. Differences between curves were evaluated using the log-rank test.

For the regression analysis, death was considered as the event, and cut-points used for *V̇*O_2peak_ and 6MWD were adopted as previously described for patients with COPD^4,17^, however, due to the divergence in the literature to define cut-points for FEV_1_ specific for severe and very severe patients with COPD, a cut-point was defined for the sample of the present study using the ROC analysis, which revealed a FEV_1_ = 25.8% (specificity = 0.714; sensitivity = 0.833) as the optimal threshold. Thus, *Cox univariate regression* model and *multivariate regression analysis* (adjusted for thresholds of MIP 55 cmH_2_O, MEP 80 cmH_2_O, FEV_1_%pred 25.8, *V̇*O_2peak_ 14.8 ml/kg/min and 6MWD 350 m) were performed, with associations expressed as hazard ratios (HRs) and 95% confidence intervals (CI). For the multivariate regression model, the presence of multicollinearity was tested using the collinearity diagnostics tool from SPSS®, and its presence was considered when variance inflation factor (VIF) ≥ 10.

## Results

A total of 79 patients with COPD were included, and their characteristics are presented in Table 1. Total sample was mainly composed by men (65.8%) with severe or very severe respiratory obstruction (91.1%); mean age was 66±9 years. The sample was stratified between survivors (*N* = 36) followed for the entire 42 months, and non-survivors (*N* = 43) followed for ~ 28 months.

**Table 1:** Characteristics of the total sample and stratified into survivors and non-survivors. Comparisons analyzed between survivors *versus* non-survivors.

Variables	Total (*N* = 79)	Survivors (*N* = 36)	Non-survivors (*N* = 43)	*p*-value
Sex (M/F)	52/27	32/4	20/23	–
Age (years)	66.2±8.7	64.5±7.9	67.6±9	0.105
Weight (Kg)	65.4±1.8	65.8±3	62.8±3.8	0.032*
BMI (Kg/m^2^)	24.1±1.6	24.7±1.5	23.4±1.2	0.048*
Time (months)	36 [28–42]	42 [42–42]	28 [4,22–31]	< 0.001*
Medications, *n* (%)				
Anticoagulant	8 (10.1)	6 (16.7)	2 (4.6)	0.132
Antihypertensive	10 (12.7)	4 (11.1)	6 (13.9)	0.748
Beta blocker	0 (0)	0 (0)	0 (0)	–
Diuretic	15 (19)	5 (13.9)	10 (2.3)	0.391
Inhaled corticosteroids	79 (100)	36 (100)	43 (100)	–
Pulmonary function				
FVC (L)	2 [1.5–2.7]	2.7 [2.2–3.3]	1.6 [1.3–1.9]	< 0.001*
FEV_1_ (L)	0.9±0.4	1.2±0.4	0.7±0.3	< 0.001*
FEV_1_/FVC	0.4 [0.3–0.5]	0.4 [0.3–0.5]	0.4 [0.3–0.5]	0.542
FEV_1_ (%pred)	35.2 [26.3–40.3]	37.4 [29.4–46.4]	29.2 [23.1–39.4]	0.001*
MVV	31.9 [2.5–41.2]	40.5 [30.5–52.8]	22.5 [18.7–37.5]	< 0.001*
GOLD (1–2-3–4)	1/6/37/35	1/5/19/11	0/1/18/24	–
MIP (cmH_2_O)	57.3±30.7	76.9±29.8	40.9±20.2	< 0.001*
MIP pred (cmH_2_O)	96.9 [82–102.5]	102.1 [97.7–106.5]	88.3 [75.6–96.9]	< 0.001*
MIP (%pred)	59 [36.6–79.3]	74.8 [58.9–90.9]	40.3 [32.8–62.9]	< 0.001*
MEP (cmH_2_O)	82.4±39.1	90.2±29.2	61.4±21.9	< 0.001*
MEP pred (cmH_2_O)	106.2 [80.2–111.8]	111.4 [107–115.9]	83.3 [72.3–106.2]	< 0.001*
MEP (%pred)	74.5 [57.8–100.5]	89.8 [72–115.8]	66.9 [54.9–87.3]	< 0.001*
Exercise tests				
6MWD (m)	398.8±107.5	468.3±58.8	356.7±108.9	< 0.001*
* V̇*_Epeak_ (L/min)	22.8 [18.1–28.8]	28.4 [24.6–38.7]	18.9 [16.6–22.8]	< 0.001*
* V̇*O_2peak_ (L/min)	1±0.4	1.2±0.4	0.8±0.2	< 0.001*
* V̇*O_2peak_ (mlO_2_/kg/min)	14.14 [11.8–17.6]	16.8 [15–19.8]	12.8 [10.6–14.5]	< 0.001*
* V̇*_E_/*V̇*CO_2slope_	30.4±5.9	27.1±3.5	33.1±6.1	0.012*
O_2_pulse (mL/beat)	8.6±2.5	9.7±2.5	7.7±2.2	< 0.001*
* V̇*_E_/MVV	1.4±0.5	1.4±0.5	1.4±0.4	0.952
* V̇*_E_/MVV (%)	130.8 [109.2–161.3]	130.5 [110.3–157.4]	130.8 [108.2–165.1]	0.764
OUES	1.5±0.8	1.7±0.9	1.4±0.7	0.236
RER	1.0±0.1	1.1±0.1	1±0.1	0.032*
HRpeak	116 [102–128]	124.5 [14.5–132]	107 [97–122]	< 0.001*
SBPpeak	170.9±28.1	190±27.1	159.4±21.9	< 0.001*
DBPpeak	89.6±10.6	95.8±11	85.9±8.6	< 0.001*
CP (mmHg.mlO_2_.min^−1^)	2582±1066	3417.6±1136.3	2076.8±614.6	< 0.001*
VP (mmHg)	5.6 [4.5–7.2]	7.5 [6.6–8.3]	5 [4.1–5.7]	< 0.001*
Borg peak (MMII)	6±2	6.4±2.9	5.6±1.3	0.061
HRR1’	107 [92–116]	113.5 [100.2–119.5]	98 [84–114]	0.022*

BMI: body mass index; FCV: forced vital capacity; FEV_1_: forced expiratory volume in one second; GOLD: classification for COPD obstruction based on the GOLD Statement; MIP: maximum inspiratory pressure; MEP: maximum expiratory pressure; 6MWD: six-minute walking distance; *V̇*_Epeak_: peak ventilation; *V̇*O_2peak_: peak oxygen output; *V̇*_E_/*V̇*CO_2slope_: ventilatory efficiency; MVV: maximum voluntary ventilation; OUES: oxygen uptake efficiency; RER: respiratory exchange ratio; HRpeak: peak heart rate; SBPpeak: peak systolic blood pressure; DBPpeak: peak diastolic blood pressure; CP: circulatory power; VP: ventilatory power; HRR1’: heart rate recovery in the first minute; M: male; F: female; Kg: kilograms; L: liters; pred: predicted; %pred: percent of predicted; cmH_2_O: centimeters of water; L/min: liters per minute; ml/Kg/min: milliliters per kilogram per minute; MMII: rating of perceived exertion in legs; *p < 0.05; non-paired *Students’ t-test*—for parametric data—and *Mann–Whitney* test—for non-parametric data—for survivors *versus* non-survivors analysis. Data are presented as absolute values, percentage, mean±SD for parametric data and median [IR 25–75%] for non-parametric data.

Non-survivors presented lower values for PF, RMS (MIP and MEP), and 6MWD, and a slightly lower BMI than survivors, despite both groups being eutrophic^18^. Regarding CPET, lower *V̇*_Epeak_, *V̇*O_2peak_, O_2_pulse, RER, peak heart rate (HRpeak), peak systolic (SBPpeak) and diastolic (DBPpeak) blood pressures, circulatory power (CP = *V̇*O_2peak_ ml/kg/min x SBP), ventilatory power (VP = SBP/*V̇*_E_/*V̇*CO_2slope_) and heart rate recovery in the first minute post-exercise (HRR1’), as well as higher *V̇*_E_/*V̇*CO_2slope_ was observed in non-survivors, all reflecting poorer exercise capacity, chronotropic response and ventilatory efficiency.

**Figure 1:**
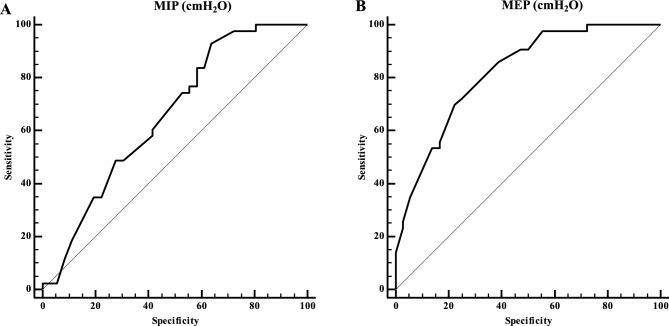
Receiver operating curves in patients with COPD for MIP (panel (**A**)—cut-off ≤ 55 cmH_2_O; AUC = 0.692; sensitivity = 60; specificity = 58) and MEP (panel (**B**)—cut-off ≤ 80 cmH_2_O; AUC = 0.736; sensitivity = 86; specificity = 61).

Cut points for RMS were analyzed considering the best sensitivity and specificity. Figure 1A shows the ROC curve for MIP (cut-off ≤ 55 cmH_2_O, sensitivity = 60 and specificity = 58) and Fig. 1B shows the ROC curve for MEP (cut-off ≤ 80 cmH_2_O, sensitivity = 86 and specificity = 61).

**Figure 2:**
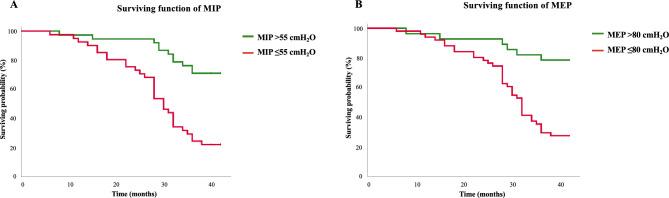
Kaplan–Meier curves for surviving probability according to the presence of MIP and MEP above and below the cut-off points found in this study (≤ 55cmH_2_O and ≤ 80cmH_2_O, respectively) over a period of 42 months (log rank for MIP: *X*^2^ = 21; df = 1; *p* = 0.000—log rank for MEP: *X*^2^ = 16; df = 1; *p* = 0.000).

**Figure 3:**
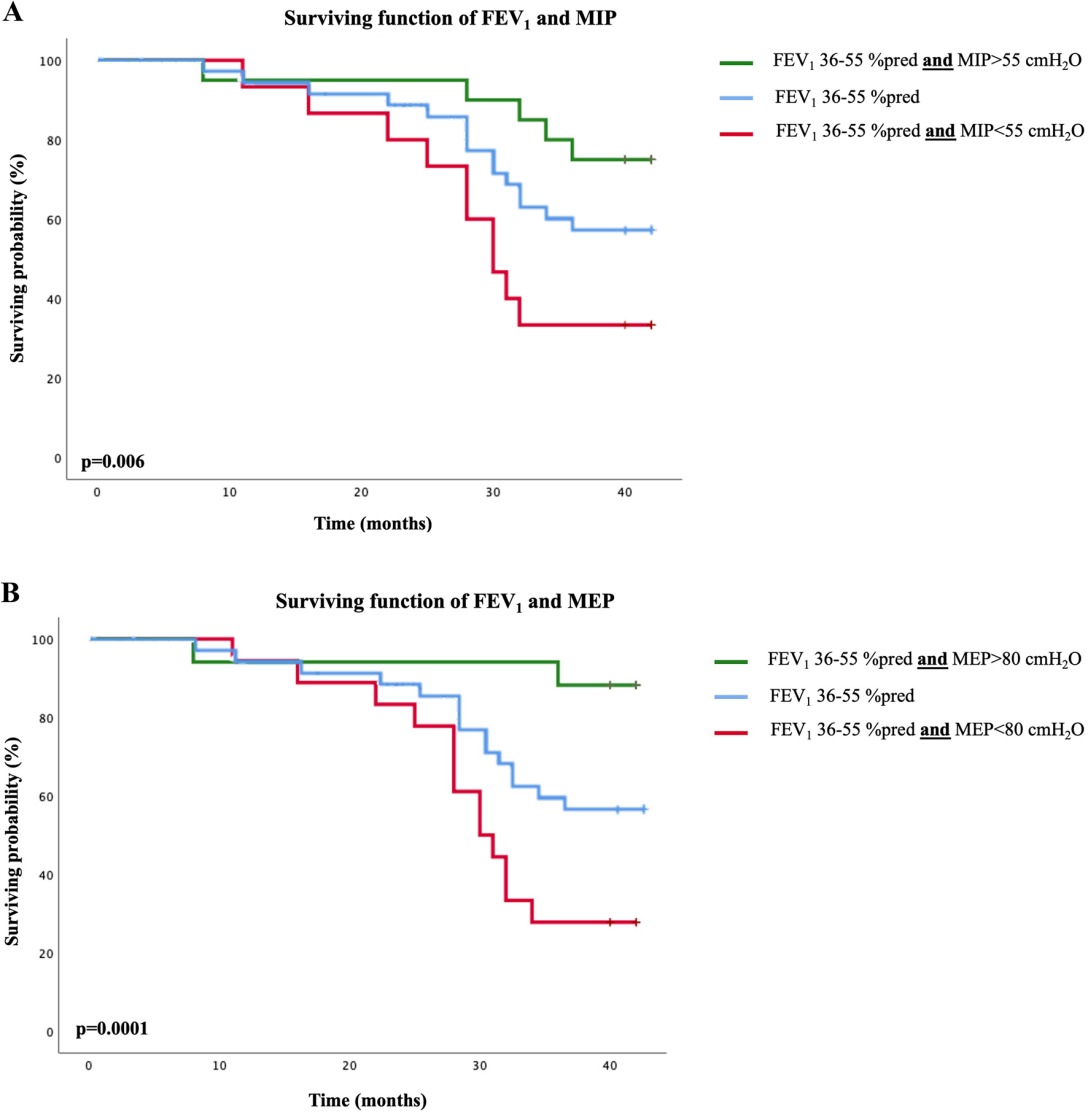
Kaplan–Meier analysis of the surviving function of FEV_1_ associated to the RMS. 3A illustrates the surviving function of *V̇*O_2peak_ and MIP (log-rank *p* = 0.006) and 3B illustrates the surviving function of *V̇*O_2peak_ and MEP (log-rank *p* < 0.001).

**Figure 4:**
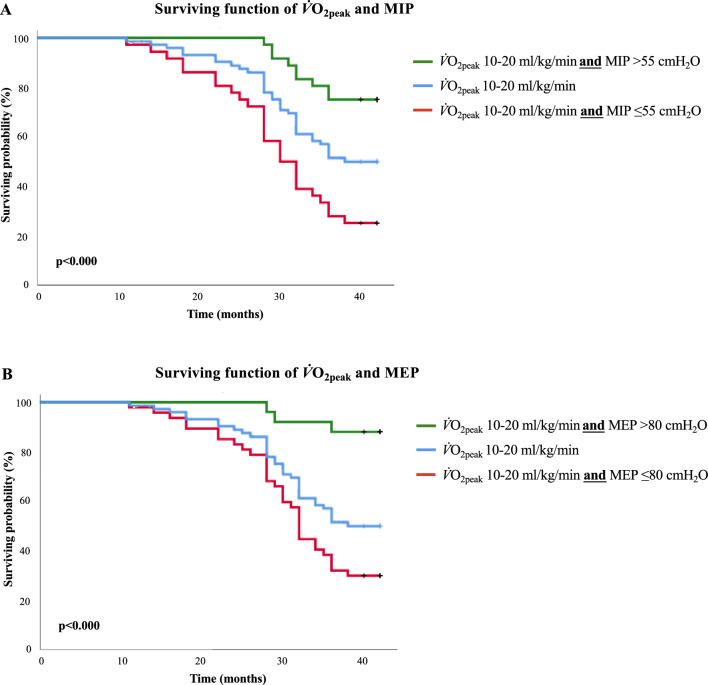
Kaplan–Meier analysis of the surviving function of *V̇*O_2peak_ associated to the RMS. 4A illustrates the surviving function of *V̇*O_2peak_ and MIP (log-rank *p* < 0.000) and 4B illustrates the surviving function of *V̇*O_2peak_ and MEP (log-rank *p* < 0.000).

**Figure 5:**
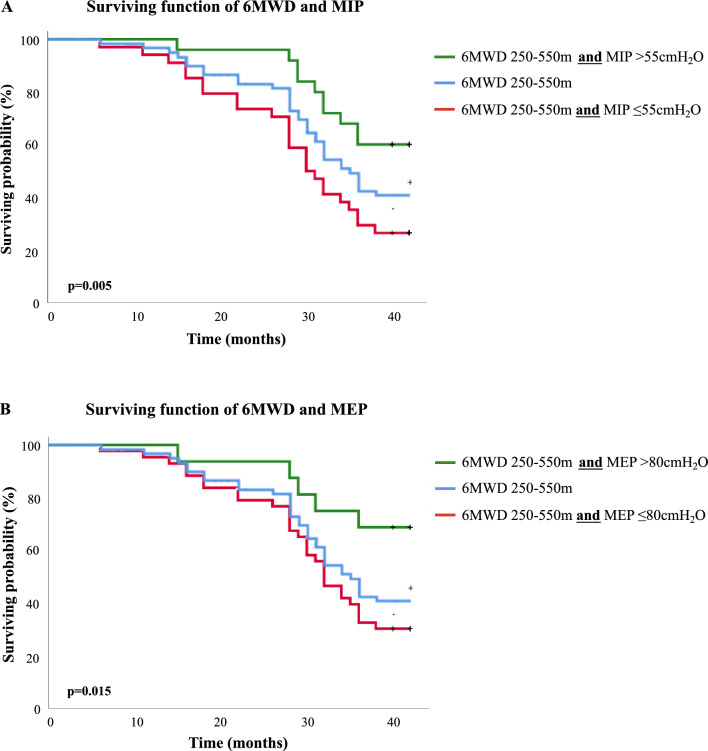
Kaplan–Meier analysis of the surviving function of 6MWD associated to the RMS. 5A illustrates the surviving function of 6MWD and MIP (log-rank *p* < 0.005) and 5B illustrates the surviving function of 6MWD and MEP (log-rank *p* < 0.015).

Survival functions derived from the Kaplan–Meier analysis considering the cut-points found for both MIP and MEP are shown in Fig. 2. The mortality curves differed significantly in the log-rank test for both MIP (*X*^2^ = 21; df = 1; *p* < 0.001) and MEP (*X*^2^ = 16; df = 1; *p* < 0.001). In addition, when combining optimal cut points of both MIP and MEP with FEV_1_ (Fig. 3A: log-rank for FEV_1_ combined with MIP *p* = 0.006; B: log-rank for FEV_1_ combined with MEP *p* = 0.0001), *V̇*O_2peak_ (Fig. 4A,B: log-rank for *V̇*O_2peak_ combined with both MIP and MEP *p* < 0.001) and 6MWD (Fig. 5A: log-rank for 6MWD combined with MIP *p* < 0.005; B: log-rank for 6MWD combined with MEP *p* < 0.015), an improvement in risk identification for mortality was observed.

The univariate Cox regression analysis is presented in Table 2 and HRs greater than 1 indicated that mortality had higher probability to happen over time in patients below the thresholds established for MIP, MEP, FEV_1_, *V̇*O_2peak_ and 6MWD, as also supported by the Kaplan–Meier survival analysis. The multivariate regression analysis for the same variables is presented in Table 3, and resulted in a statistically significant model [F(5,62) = 11.590; *p* < 0.001; R^2^ = 0.483], however, MIP ≤ 55 cmH_2_O (*β* = 0.387; *t* = 3.410; *p* = 0.001), MEP ≤ 80 cmH_2_O (*β* = 0.225; *t* = 2.159; *p* = 0.035); *V̇*O_2peak_ 14.6 ml/Kg/min (*β* = 0.436; *t* = 4.430; *p* < 0.001) and 6MWD 350 m (*β* = 0.214; *t* = 2; *p* = 0.049) were identified as predictors for mortality, which was not observed for FEV_1_ 25.8%pred (*β* = 0.125; *t* = 1.343; *p* = 0.184).

**Table 2:** Cox univariate regression analysis of the risk factors for mortality in 79 patients with COPD.

Covariates	Coefficient	Standard error	Hazard ratio (CI 95%)	*p-value*
MIP ≤ 55 cmH_2_O	1.458	0.353	4.296 (2.151–8.580)	< 0.001*
MEP ≤ 80 cmH_2_O	1.579	0.442	4.848 (2.037–11.540)	< 0.001*
FEV_1_ ≤ 25.8%pred	0.347	0.342	1.415 (0.724–2.765)	0.310
*V̇*O_2peak_ ≤ 14.6 ml/Kg/min	1.620	0.366	5.054 (2.466–10.356)	< 0.001*
6MWD ≤ 350 m	1.950	0.341	7.029 (3.604–13.710)	< 0.001*

MIP: maximum inspiratory pressure; MEP: maximum expiratory pressure; FEV_1_: forced expiratory volume in the first second; *V̇*O_2peak_: peak oxygen uptake; 6MWD: six-minute walking distance; cmH_2_O: centimeters of water; %pred: percent of predicted; ml/Kg/min: milliliters per kilogram per minute; m: meters. Univariate Cox regression analysis; *p < 0.05.

**Table 3:** Multivariate regression analysis of the risk factors for mortality in 79 patients with COPD.

Model	Standardized coefficients	*t*	*p-value*	Collinearity diagnosis
	*β*			VIF
5				
MIP ≤ 55 cmH_2_O	0.387	3.410	0.001*	1
MEP ≤ 80 cmH_2_O	0.225	2.159	0.035*	1.303
FEV_1_ 25.8%pred	0.125	1.343	0.184	1.035
*V̇*O_2peak_ 14.6 ml/Kg/min	0.436	4.430	< 0.001*	1.160
6MWD 350 m	0.214	2.0	0.049*	1.376

MIP: maximum inspiratory pressure; MEP: maximum expiratory pressure; FEV_1_: forced expiratory volume in the first second; *V̇*O_2peak_: peak oxygen uptake; 6MWD: six-minute walking distance; cmH_2_O: centimeters of water; ml/Kg/min: milliliters per kilogram per minute; m: meters; VIF: variance inflation factor. Multivariate regression analysis; *p < 0.05.

## Discussion

This study focused on investigating RMS and its role in predicting mortality in patients with COPD, as well as its contribution to improve risk identification when combined with previously established prognostic measures for this population. However, when stratifying the patients, some clinical differences between survivors and non-survivors became evident, and it is important to discuss their clinical differences in a general context, which can improve the clinical management of the disease.

A high mortality rate was observed in our sample, and it is important to highlight that both groups were mostly composed by patients with GOLD III and IV, i.e., severe and very severe patients. However, non-survivors presented worse PF and RMS, which were previously established as predictors of mortality in the overall population^19^ and in COPD^20,21^, as well as lower maximum voluntary ventilation (MVV) and 6MWD. MVV was previously found to correlate equally or better than FEV_1_ with outcomes of dyspnea and exercise capacity in COPD, as well as correlations were observed between MVV and 6MWD^22^. Also, the association between 6MWD and risk of death was previously established^23^, with 6MWD ≤350 m being associated with increased risk of hospitalizations, exacerbations, and death.

CPET data revealed lower exercise capacity in non-survivors. Recently, Ewert et al.^4^ found a lower survival probability in patients with lower *V̇*_Epeak_, *V̇*O_2peak_, and poor ventilatory efficiency (i.e., higher *V̇*_E_/*V̇*CO_2slope_), consistent with non-survivors in the present study. Lower *V̇*O_2peak_ is an independent predictor of death in COPD^24^, and its association with lower FEV_1_ was confirmed in previous studies^25^. Higher *V̇*_E_/*V̇*CO_2slope_ has been also shown as a predictor of mortality in COPD^26^, and other studies demonstrated the relation between poor ventilatory efficiency and its negative association with survival in COPD and HF^27–29^. Furthermore, previous findings observed that O_2_pulse tends to decline in conjunction with worsening PF in COPD^30^, being a predictor of all-cause mortality in patients with coronary artery disease (CAD)^31^.

Non-survivors presented worse chronotropic response (lower HRpeak, SBPpeak, DBPpeak, CP, VP and HRR1’), which has been previously associated with severe COPD^32^, and an independent predictor of adverse events^33^. Our findings also corroborate to Hulo et al.^34^, who found that the worse the PF, the lower the peak HR and blood pressure. Moreover, although little is known regarding CP and VP and its ability to predict prognosis in COPD, these variables have been investigated in patients with HF, and its lower values were previously reported in patients with CAD, predicting risk of hospitalization and death^35^.

Although both survivors and non-survivors presented low MIP and MEP when compared to the established values for healthy subjects^15^, lower values were observed in non-survivors. Because respiratory muscles play an important role in the mechanics of ventilation^36^ and their structure and function are impaired in COPD^37^, other outcomes are possible.

In a retrospective cohort study, Rodrigues et al.^3^ found inspiratory muscle strength to be a powerful discriminatory measure to identify risk of death in patients with COPD and reported MIP < 73cmH_2_O and 6MWD < 479 m to be optimal cut-points for increased risk of death in 2 years, even though a cut-point for expiratory muscle strength was not evaluated. In their study, the sample was mostly composed of patients with GOLD II and III, and our study was mainly composed of patients with GOLD III and IV, for whom MIP < 55cmH_2_O was identified as a cut-off point for mortality in 42 months.

In addition, regarding patients with severe and very-severe airflow limitation, our results corroborate those of Kim et al.^9^, who found means of 53.2 and 83.7 cmH_2_O for MIP and MEP, respectively. Also, despite the influences of COPD on MEP^9^, in a systematic review with meta-analysis^10^ assessing the effects of expiratory muscle training (EMT) and EMT plus inspiratory muscle training, EMT alone was reported to contribute to increases in both MEP and MIP, supporting this type of training as part of the treatment in patients with severe and very severe COPD.

Furthermore, in terms of the contribution of RMS to improve the prognosis assessment when combined to other predictors of mortality, substantial differences in identifying patients at higher risk were observed when associating MIP and MEP with FEV_1_, *V̇*O_2peak_ and 6MWD. In this regard, a reduced FEV_1_ was previously strongly associated with poor prognosis in patients with COPD, showing that the lower the FEV_1_, the worse the prognosis^2^. Furthermore, Meyer et al.^11^ found MIP as an independent predictor of prognosis in HF, indicating that its association with *V̇*O_2peak_ can improve risk stratification, since patients with *V̇*O_2peak_ between 10–20 ml/kg/min and MIP < 5.3 kPa had worse prognosis at 36 months. The authors highlighted MIP as an additional measure to improve risk stratification for patients with HF when combined with *V̇*O_2peak_. Since we are dealing with COPD in the present study, which is similarly a persistent and progressive disease usually associated with worse prognosis, our results indicate: (1) MIP and MEP are valuable predictors of mortality in COPD; and (2) risk assessment assessed through *V̇*O_2peak_ and 6MWD is improved when combining with RMS. Moreover, the interaction between static functional parameters (MIP and MEP) associated with dynamic factors (*V̇*O_2peak_, 6MWD, among other dynamic functional variables) and its influence in the survival probability of patients severely and very severely affected by COPD is still a poorly investigated phenomenon. It is important to highlight that the GOLD Report of 2023^1^ recommends inspiratory and expiratory muscle strength to be included as important assessments in rehabilitation programs. This points out the importance of assessing and training both inspiratory and expiratory muscles as part of the COPD management.

Our study has some limitations that should be stated. As a retrospective cohort study, previous data about the comorbidities of the patients included as well as other lung function variables are not available to be considered in the analysis. Also, having a greater number of patients in our sample could better respond to the questions, especially giving a stronger sensitivity to the cut-off points found. In this regard, is should also be highlighted that the follow-up period of this study is higher than most of the studies in the literature with this population, added to the fact that our sample is mostly composed by severe and very patients with COPD, which represents a population usually difficult to assess in terms of acceptance and adherence to the proposed protocols. However, as a progressive disease with one of the highest mortality rates worldwide, this study addresses a very important topic to improve strategies to identify patients with COPD with increased risk of death, using accessible assessments (MIP and MEP) to be added to evaluations usually performed (CPET and 6MWT) in research and clinical/rehabilitation settings.

## Conclusion

Patients severely affected by COPD presenting MIP ≤ 55 and/or MEP ≤ 80 cmH_2_O are at increased risk of mortality. Furthermore, MIP and MEP substantially improve the mortality risk assessment when combined with FEV_1_, *V̇*O_2peak_ and 6MWD in patients with COPD. Thus, because assessing RMS is easy and applicable in clinical practice, MIP and MEP cut points found in this study can be useful especially for patients severely affected by COPD, helping to improve risk evaluation and identification in terms of patients’ eligibility criteria for urgent pharmacological optimization, pulmonary rehabilitation, and lung volume reduction surgery.

### Supplementary Information

Supplementary Figure 1.

## Data Availability

The datasets used and analyzed during the current study are available from the corresponding author on reasonable request.
