# Supplementary material for: Respiratory muscle strength can improve the prognostic assessment in COPD
Source: Sci Rep. 2024 May 29;14:12360. doi: 10.1038/s41598-024-54264-w (PMC11137089; doi:10.1038/s41598-024-54264-w)
Supplement: Supplementary file 1 — Supplementary Figure 1. [file 41598_2024_54264_MOESM1_ESM.pdf]

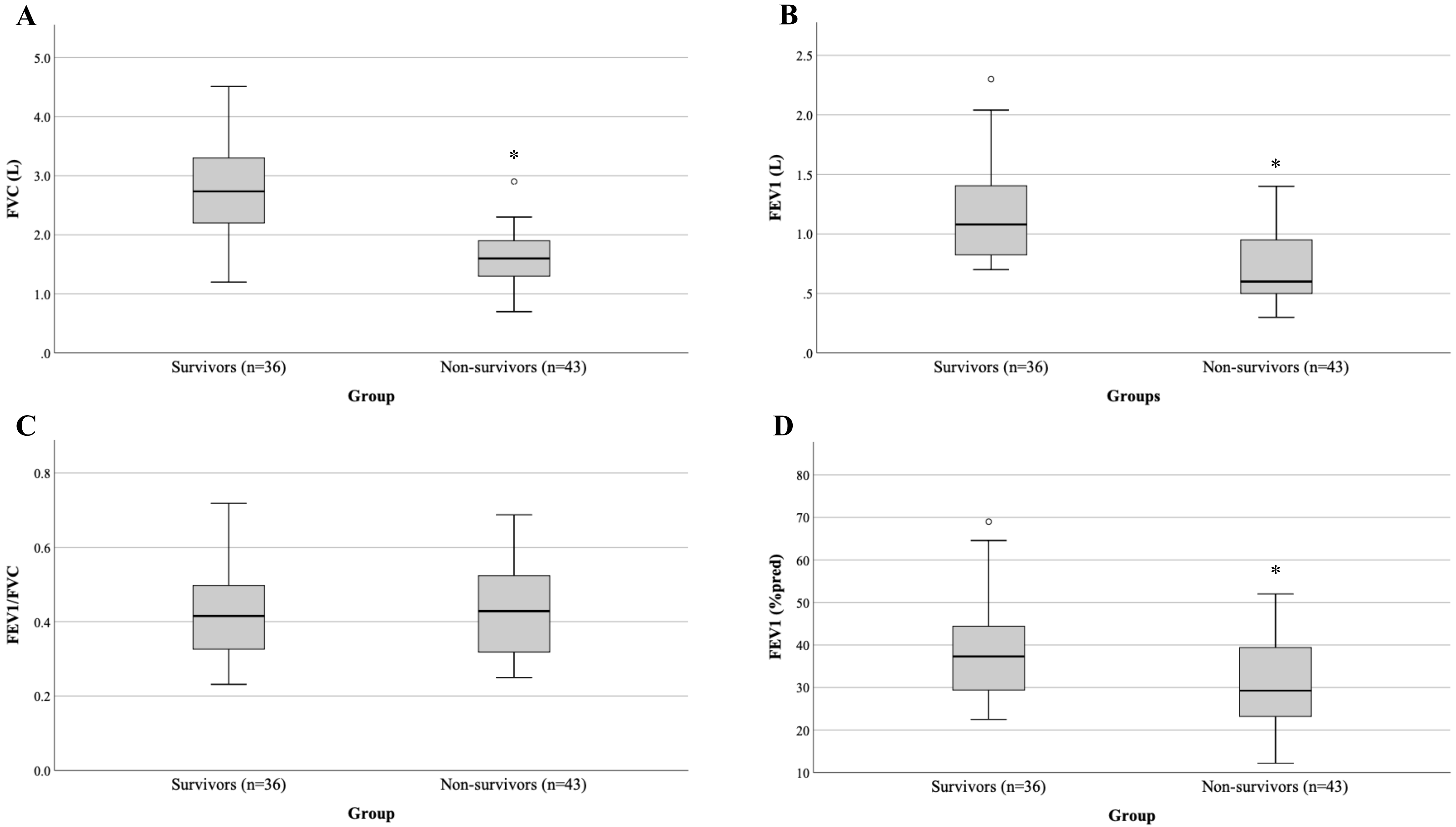

Supplementary Figure 1: Diagram in boxplot graphs presenting the pulmonary function variables of the sample stratified between survivors and non-survivors. Panel A: forced vital capacity (FVC); Panel B: forced expiratory volume in the first second (FEV1); Panel C: FEV1/FVC ratio; Panel D: forced expiratory volume in the first second as percent of predicted. *: $p<0.05$ when comparing survivors *versus* non-survivors.
